# Positive Effects of (+)-Epicatechin on Traumatic Spinal Cord Injury Recovery

**DOI:** 10.3390/biom15060869

**Published:** 2025-06-14

**Authors:** Cristian Gonzalez-Ruiz, Rodrigo Mondragón-Lozano, Hermelinda Salgado-Ceballos, Francisco Villarreal, Yuridia Martínez-Meza, Eduardo Meaney, Nayelli Nájera, Guillermo Ceballos

**Affiliations:** 1 Laboratorio de Investigación Integral Cardiometabólica, Escuela Superior de Medicina, Instituto Politécnico Nacional, Mexico City 11340, Mexico; adrian_g_90@hotmail.com (C.G.-R.); yurimtz@live.com.mx (Y.M.-M.); emeaneym@ipn.mx (E.M.); 2 Facultad de Estudios Superiores Iztacala, Universidad Nacional Autonoma de México, Tlalnepantla 54090, Mexico; 3 Unidad de Investigación Médica en Enfermedades Neurológicas, Hospital de Especialidades Centro Médico Nacional Siglo XXI, Instituto Mexicano del Seguro Social, Mexico City 06600, Mexicomelisalce@yahoo.com (H.S.-C.); 4 School of Medicine, University of California San Diego, San Diego, CA 92093, USA

**Keywords:** spinal cord injury, (+)-epicatechin, moderate spinal trauma

## Abstract

Neurological damage from traumatic spinal cord injury (SCI) results in a grade of disability ranging from mild to severe motor and sensory dysfunction. It occurs more frequently in men of productive age. Treatment essentially consists of anti-inflammatories and rehabilitation. Other treatments are only partially effective, and inadequate treatment and secondary conditions often cause premature mortality. The search for pharmacological approaches is a continuous effort. This study aimed to assess the effects of a natural compound on spinal cord injury (SCI) as an alternative damage prevention maneuver. We evaluated the protective effects of the flavanol (+)-epicatechin (EC) in a rat model of moderate trauma-induced SCI on protein markers of damage events. The results showed that EC induced significant protection against SCI. No changes were found in angiopoietin-1, beclin-1, myelin basic protein, glial fibrillary acidic protein, neurofilament heavy polypeptide, and neuronal nuclear antigen after the injury, suggesting that damage progression was impeded. The reduction in damage translates into better movement. The results suggest that (+)-epicatechin may be a suitable alternative for treating SCI.

## 1. Introduction

A traumatic spinal cord injury (SCI) can be caused by various incidents, such as car accidents, falls, sports injuries, or acts of violence, impacting mobility, sensation, and quality of life. It occurs when the spinal cord is damaged by a sudden impact, resulting in partial or complete loss of function below the level of the injury. The degree of impairment depends on the severity of the injury and its location in the spinal cord. SCI causes complete or incomplete loss of sensory and motor functions below the level of injury [1,2,3], many comorbidities such as bladder and bowel dysfunction, obesity, diabetes, neuropathic pain, muscle atrophy, and other associated consequences such as psycho-social problems [1]

It is estimated that the annual incidence of SCI in Mexico is 18.1 per million inhabitants [2]. In the United States (US), each year, about 11,000 individuals suffer from SCI, and the annual cost of treating patients who do not die immediately approaches USD 11 billion (https://www.cdc.gov/health-topics.html, accessed date, 11 July 2024).

In this sense, many people worldwide live with SCI; approximately 20.6 million individuals were affected in 2019 [3].

SCI care includes spinal support and stabilization treatments; most subjects require a decompression or surgical stabilization procedure. Almost all individuals receive anti-inflammatory treatment. Once the acute phase is over, rehabilitation is provided. However, inadequate treatment and secondary conditions often cause premature mortality.

SCI represents a significant public health problem. Since current treatments are only partially effective, a considerable research effort is necessary to explore alternative approaches that are safe and free of secondary effects [4,5,6,7,8,9,10,11].

In this sense, many phytochemical compounds have been tested. In this study, we explore (+)-epicatechin, which is part of the catechins, a large family of secondary metabolites found in various vegetable products, such as cacao, green tea, and guarana. This large family of biomolecules is known as flavonoids. The subclass known as flavanols comprises four stereoisomers: (−)-epicatechin, (+)-epicatechin, (−)-catechin, and (+)-catechin.

**Figure 1:**
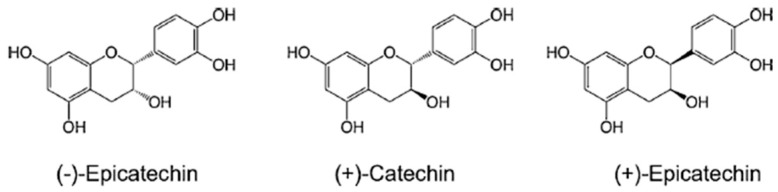
Chemical structures of (−)-epicatechin and its isomers (+)-catechin and (+)-epicatechin.

The molecular formula (C_15_H_14_O_6_) of these substances is similar. These flavanols differ only in their spatial structure (−)-epicatechin ((2*R*,3*R*)-2-(3,4-dihydroxyphenyl)-3,4-dihydro-2*H*-chromene-3,5,7-triol) and (+)-epicatechin ((2*S*,3*S*)-2-(3,4-dihydroxyphenyl)-3,4-dihydro-2*H*-chromene-3,5,7-triol) (Figure 1).

Several studies have shown that (−)-epicatechin induces beneficial effects on the skeletal muscle and the cardiovascular system, including damage to the skeletal muscle’s sarcomeric structure and mitochondrial malfunction by promoting mitochondrial biogenesis [12,13,14].

The effects are associated with the activation of intermediaries in specific signaling pathways, such as the MAPKs, Akt, and AMPK, which depend on transmembrane receptor activation. We have shown that (−)-epicatechin selectively binds to the apelin receptor, inducing many of its effects through receptor activation [15].

Interestingly, the stereoisomer (+)-epicatechin was more active than (−)-epicatechin in stimulating mitochondrial biogenesis and mitochondria-related endpoints in cultured cells and mice [16,17,18].

Recently, we have demonstrated that (−)-epicatechin reduces muscle atrophy and loss after complete spinal cord injury (SCI) by transection [19], suggesting a possible modulation of damage in extreme spinal cord injury.

Based on the reported differences in potency, this work analyzed the effects of (+)-epicatechin in a model of moderate traumatic spinal cord injury and explored its impact on several aspects of continuous spinal cord damage following the contusion. We hypothesized that this flavanol would protect the spinal cord against trauma-induced damage.

In this study, we investigated the neuroprotective potential of the flavanol (+)-epicatechin (EC) by evaluating its effects on several key molecular markers associated with spinal cord injury (SCI). Among these, angiopoietin-1 has been reported to exert neuroprotective effects by promoting vascular stability and neuronal survival following injury [20]. We also examined myelin basic protein (MBP), a critical structural component of the myelin sheath that plays a pivotal role in axonal protection and regeneration [21]. To assess neuronal integrity, we analyzed the expression of neuronal nuclei antigen (NeuN), a widely used marker for mature neurons, which serves as an indicator of neuronal presence, loss, or regeneration in both physiological and pathological conditions [22]. Additionally, glial fibrillary acidic protein (GFAP) was evaluated as a marker of astrocyte reactivity and gliosis, processes known to intensify following SCI [23]. Lastly, we assessed neurofilament heavy chain (NFH), a key component of the neuronal cytoskeleton whose altered expression reflects axonal damage and degeneration after central nervous system injuries [24]. Through this panel of markers, we aimed to comprehensively evaluate the cellular and molecular impact of EC on spinal cord pathology.

## 2. Materials and Methods

### 2.1. Ethical Approval

This study was approved by the institutional committee (ESM-Cicual-01/22-05-2018) and followed the guidelines of Mexican Official Standard NOM-062-ZOO-1999. Institutional research and ethics committees approved the protocol. Procedures and technical specifications for the production and animal care complied with the recommendations of the Guide for the Care and Use of Laboratory Animals of the National Institutes of Health (Institute of Laboratory Animal Resource (US) Committee on Care and Use of Laboratory Animal 2011). A total of 32 adult female Long Evans rats, weighing between 300 and 400 g, were obtained from Instituto Camina AC, housed in a controlled environment, and provided with water and standard rodent chow ad libitum. Female rats are commonly used in SCI models, as they exhibit better outcomes following spinal cord injury due to their enhanced functional recovery, greater preservation of neural tissue, and less aggressive inflammatory response [25].

### 2.2. Surgical Procedure

The animals were anesthetized using a ketamine/xylazine cocktail intramuscular (90 mg/kg Anestek^®^ PISA (Pisa Farmacéutica, CDMX, México), 6 mg/kg Procin ^®^ PiSA). A longitudinal incision was made in the vertebral zone (T8 to T10). Paravertebral muscles were sectioned, exposing T9 vertebrae; subsequently, a laminectomy was performed. Once the spine was exposed, a moderate traumatic spinal cord injury was performed with an impact of moderate contusion (using a stainless-steel bar of 10 g and 2 mm diameter dropped from 25 mm height) using a New York Impactor. Subsequently, the tissue layers were sutured, and the animals were transferred to a warm environment for recovery. All procedures followed the proposals of Renuka Verma et al., with minimal variations [26].

### 2.3. Post-Surgical Care

Surgical aftercare was performed using the previously reported methodology of Ramsey et al. [27]. The first three days after surgery, experimental animals were administrated with non-steroidal anti-inflammatory ketoprofen (Ketofen^®^ CEVA) 5 mg/kg/day and the antibiotic drug enrofloxacin (Baytril^®^ BAYER) 10 mg/kg/day subcutaneously; the animal bladders were manually emptied thrice a day during the first 15 days and subsequently once a day until the experimental period ended. The animals’ health was verified daily throughout all experimental periods, and primary care was provided in cases of illness or lesions, such as auto-sarcophagy of limbs, edema, or spasticity.

### 2.4. Study Design

After the surgery, animals were randomly assigned to one of the following groups and followed for 21 days:

(1) Sham group: Group with laminectomy without moderate contusion (*n* = 8);
(2) Control group: Laminectomy + moderate contusion + administration of water as a vehicle [1 mL/kg of body weight/day] by oral gavage (*n* = 12 rats);
(3) Treated group: Laminectomy + moderate contusion + administration of (+)-epicatechin in water [1 mg/mL/kg of body weight/day] by oral gavage (*n* = 12 rats).

**Figure 2:**
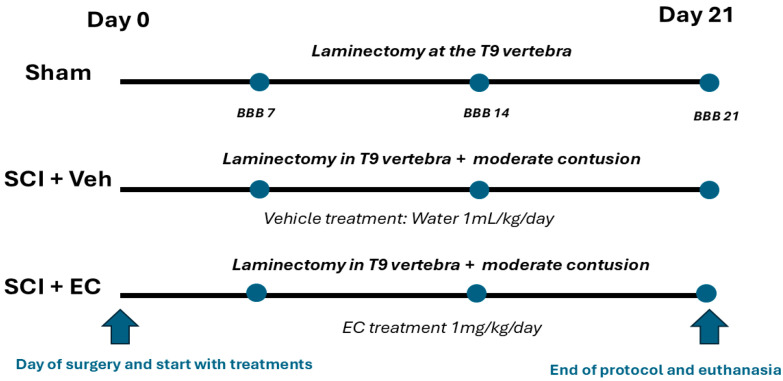
Experimental setup scheme: Female Long Evans rats underwent laminectomy at T9 followed by moderate contusion injury. Animals were randomized into three groups: sham (laminectomy only, *n* = 8), control (injury + vehicle, *n* = 12), and (+)-EC-treated (injury + 1 mg/kg/day (+)-epicatechin, *n* = 12). Treatments were administered via oral gavage starting 24 h post-injury. Functional recovery (BBB scale) was assessed at 7, 14, and 21 days. Spinal cord tissue was collected at the endpoint (day 21) for protein analysis.

Treatments were initiated one day after the surgery, and the dose was based on previous results [28,29]. A mean-difference unpaired t-test was used to calculate the sample size (*n* = 7) and statistical power of the study (0.831). (+)-Epicatechin was a gift from Epirium Inc. (San Diego, CA, USA). The experimental timeline and group assignments are summarized in Figure 2.

### 2.5. Functional Recovery Analysis

The functional recovery test was based on previous reports by Basso et al. and Koopman et al. [30,31]. Limb motor function was assessed using the Basso, Beatie, and Bresnahan (BBB) locomotion scale, which consists of 21 points, where a value of 0 points indicates the absence of movement, and 21 points indicate normal gait equal to that of non-spinal-cord-injured rats [30]. The evaluation was performed 7, 14, and 21 days after spinal cord injury (SCI) in an open-field box using a double-blind scheme and was assessed by three independent, trained evaluators.

### 2.6. Tissue Collection

After the experiment protocol ended, animals were euthanized using pentobarbital sodium (PISABENTAL ^®^ PiSA 100 mg/kg) by intraperitoneal injection. Subsequently, the spinal cord was collected, considering 1 cm above the epicenter of the lesion to the rostral region and 1 cm below the epicenter of the lesion. The spinal cord tissue was immediately frozen at −80 °C.

### 2.7. Western Blot

We used a GAPDH Western blot for a linearity test, loading the protein sample with 10–70 μg. All Western blots were performed using a 30 μg loading sample. Western blots were performed in samples from three randomly chosen animals per group.

We followed standard methodologies for Western blot assays; briefly, 50 mg of spinal cord tissue was homogenized, 400 µL lysis buffer containing HEPES 50 mM with protease and phosphatase inhibitors (P8340 Sigma-Aldrich and P5726 Sigma Aldrich, respectively) was added, EDTA 1.3 mM and EGTA 1 mM were used as chelating agents (pH 7.5), tissue homogenates were sonicated for 20 min at 4 °C and then centrifuged 13,000 rpm at 4 °C for 10 min, and the supernatant was isolated. The total protein content of the supernatant was measured using Bradford reagent (BIO-RAD, Mexico City, México).

Proteins were boiled and then loaded into a 4% stacking gel and a 10% separating SDS–polyacrylamide gel. They were separated using electrophoresis with a variable voltage. Then, they were transferred to PVDF membranes (Immuno-blot ^®^ BIO-RAD, México).

Transferred membranes were treated with blocking buffer (5% non-fat dry milk in TBS + Tween 1%) for 1 h at room temperature, followed by three 5 min TBS-T washes each. Subsequently, a primary antibody was added, and the membrane was incubated overnight at 4 °C. After this step, the membranes were washed three times with TBS-T for 5 min each, then incubated with a secondary antibody for one hour at room temperature.

Finally, the membranes were washed three times in TBS-T for five minutes each. The PVDF membranes were then immunodetected using a luminol reagent (Santa Cruz Biotechnology, Dallas, TX, USA) for 5 min and scanned in the LI-COR C-Digit Scan system for 12 min.

### 2.8. Antibodies

The primary antibodies used in this protocol were anti-myelin basic protein (Abcam, ab218011; 1:100), anti-angiopoietin-1 (Abcam, ab102015; 1:200), anti-beclin-1 (Abcam, ab207612; 1:100), anti-neurofilament heavy polypeptide (Abcam, ab4680; 1:250)**,** anti-NeuN (Abcam, ab177487; 1:100), and anti-GFAP (Abcam, ab237844; 1:100). The secondary antibodies were goat anti-rabbit IgG HRP-conjugated (Thermo-Fisher Scientific ^®^, Waltham, MA, USA).

### 2.9. Statistical Analysis

We analyzed the data using descriptive statistics; a normal distribution and homogeneity test of variances were applied using a Shapiro–Wilk test. Posteriorly for Western blot, we applied a one-way ANOVA or Kruskal–Wallis test depending on the normality test results, and multiple comparison Tukey’s post hoc test was applied. The statistical significance of BBB tests was assessed with two-way ANOVA with Sidak’s post hoc test. We also performed linear regression of BBB results, obtaining the slopes and comparing them between groups. The data were expressed as mean ± SEM; a *p* < 0.05 was considered statistically significant. GraphPad Prism v 10.0 was used to perform all tests.

## 3. Results

### 3.1. BBB Score

**Figure 3:**
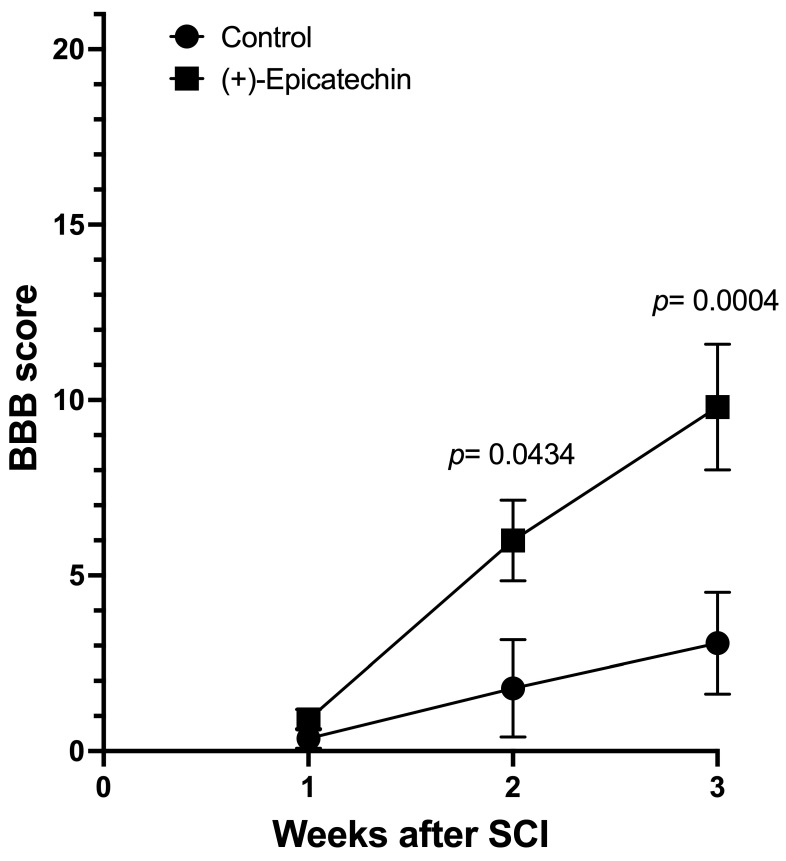
**BBB scores:** BBB scores were obtained by three blinded independent researchers. Results are expressed as mean ± SEM. Circles represent the control group (moderate contusion + vehicle, *n* = 12), and squares represent the treated group (moderate contusion plus (+)-EC, *n* = 12). Two-way ANOVA tests were performed, followed by the Sidak multiple comparisons post hoc test. Statistical differences are shown in the graph. The slopes of the changes (BBB scores/week) are significantly different (*p* = 0.014) and represent a 227.9% difference in mobility recovery in the (+)-Epi-treated group.

Figure 3 presents the analysis of the BBB score. The score is higher in the (+)-EC-treated group every week analyzed, reaching statistical significance in the second week (14 days) after the SCI. The recovery at three weeks (21 days) almost doubled the BBB value compared to the control group (rats treated with vehicle) (see Supplemental Video).

### 3.2. Protein Marker Changes

**Figure 4:**
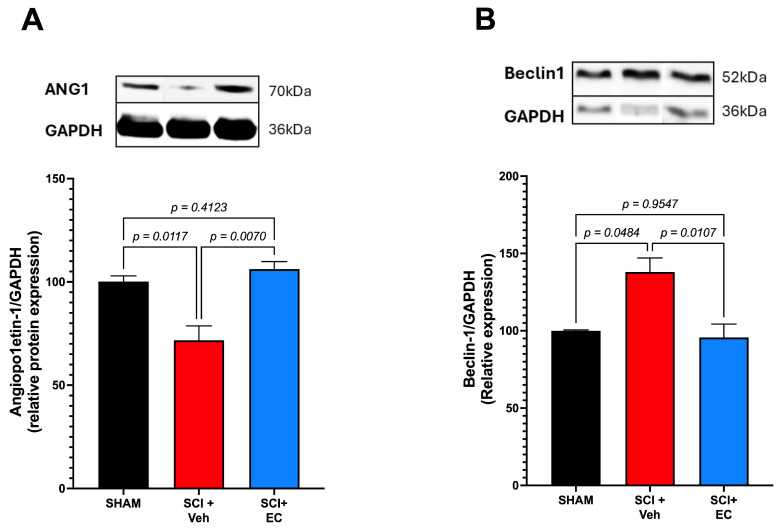
(**A**) **Relative expression of angiopoietin-1 and** (**B**) **relative expression of beclin-1:** Western blots quantified by densitometry. Results are expressed as mean ± SEM. One-way ANOVA analysis was performed, followed by multiple comparison post hoc tests. Statistical values are shown in the graph. Original Western blot images are provided in the Supplementary Materials.

The analysis of angiopoietin-1 expression as a marker of blood vessel stabilization in the spinal cord near the site of SCI is shown in Figure 4A. SCI induced a significant decrease in the relative expression of this protein. A significant difference was found between the SCI + vehicle and SCI plus (+)-EC groups. The analysis of the differences between the sham group and the SCI plus (+)-EC group shows no differences; this result suggests that (+)-EC treatment protects against the SCI-induced decrease in angiopoietin-1.

Beclin-1, a marker of neuronal damage associated with autophagy regulation, was evaluated. The results show a significant increase in beclin-1 in the rats with SCI + vehicle compared to the sham group, as shown in Figure 4B. Interestingly, no change was found in the group treated with (+)-EC compared to the sham group, suggesting that the flavanol blocks the neuronal damage associated with the increased beclin-1.

Similarly, we measured glial fibrillary acidic protein (GFAP) expressed in astrocytes, which increases after SCI and contributes to glial scar formation.

**Figure 5:**
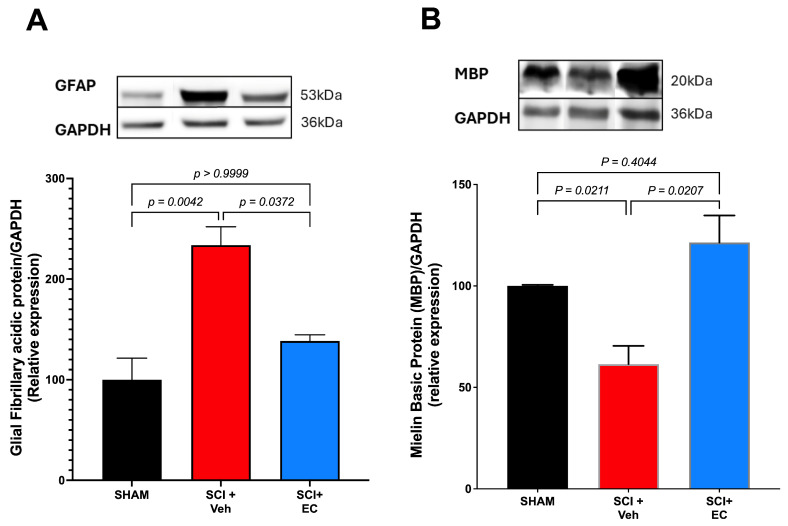
(**A**) **Relative expression of glial fibrillary acidic protein (GFAP) and** (**B**) **relative expression of myelin basic protein (MBP):** Western blots quantified by densitometry. Results are expressed as mean ± SEM. One-way ANOVA analysis was performed, followed by multiple comparison post hoc tests. Statistical values are shown in the graph. Original Western blot images are provided in the Supplementary Materials.

The results show a remarkable and statistically significant increase in the relative expression of GFAP in the SCI + vehicle group compared with the sham group. Treatment with (+)-EPI decreases the expression of GFAP compared to the SCI + vehicle group, showing no statistical differences with the sham group (Figure 5A).

We also analyzed the demyelination process to assess spinal cord injury by measuring myelin basic protein (MBP) expression. The results show a significant decrease in MBP expression in the SCI + vehicle group compared to the sham group. The group treated with (+)-EC shows a slight but not significant increase in MBP’s relative expression, suggesting a limitation of damage induced during SCI (Figure 5B).

**Figure 6:**
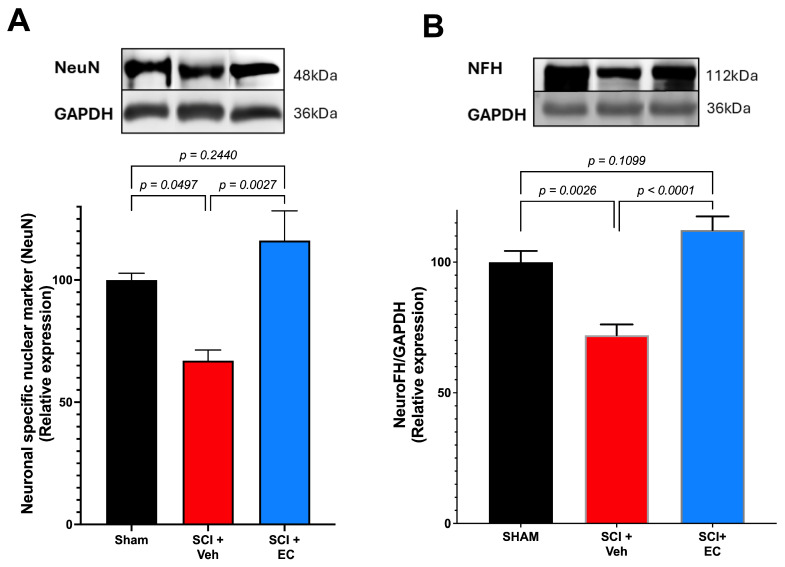
(**A**) **Relative expression of NeuN protein and** (**B**) **relative expression of neurofilament heavy polypeptide (NFH):** Western blots quantified by densitometry. Results are expressed as mean ± SEM. One-way ANOVA analysis was performed, followed by multiple comparison post hoc tests. Statistical values are shown in the graph. Original Western blot images are provided in the Supplementary Materials.

Since we found a protective effect of (+)-EC on the damage induced by traumatic SCI, we explored the expression of a neuronal-specific marker (NeuN), a nuclear protein expressed in neurons. Interestingly, as a confirmation of the effects, NeuN expression significantly decreased in the SCI + vehicle group compared to the sham group. We also found that treatment with (+)-EPI blocked the decrease in NeuN, suggesting a protective role (Figure 6A).

We also evaluate changes in neurofilament heavy chain (NFH), a structural protein of the neuron cytoskeleton and white matter tracts that is highly affected in spinal cord injury-induced neurodegeneration; our results show a significant decrease in NFH expression in the SCI + vehicle group compared to the sham group (Figure 6B). Again, treatment with EC blocks the reduction in NFH, suggesting a protective role.

## 4. Discussion

The main results found in this work showed for the first time that the flavanol (+)-epicatechin induced clear and significant protection against spinal cord injury caused by moderate trauma. Protection against damage was observed, as no changes were found in angiopoietin-1, beclin-1, myelin basic protein, glial fibrillary acidic protein, neurofilament heavy polypeptide, and neuronal nuclear antigen after the injury. The reduction in damage translates into improved movement, as measured by the BBB score.

Interestingly, the induced effects of (+)-EC treatment can prevent the progression of SCI-induced damage, resulting in improved mobility compared to the control group.

Additionally, we have demonstrated that (+)-EC is substantially more active than (−)-epicatechin in stimulating mitochondrial biogenesis, as indicated by the protein levels of electron transport chain complexes II and IV, as well as mitochondria-related endpoints [18].

Based on previous results, this work analyzed the effects of (+)-epicatechin in a model of moderate traumatic spinal cord injury [30] and explored its effects at several points of continuous spinal cord damage following the contusion.

We followed the animals for three weeks, exploring their mobility before euthanasia. We employed the BBB Scoring to assess the motor function of rats. Post-injury motor behavior was evaluated via the Basso, Beattie, and Bresnahan (BBB) locomotor scale method [30].

The scale (0–21) represents sequential recovery stages and categorizes combinations of rat joint movement, hindlimb movements, stepping, forelimb and hindlimb coordination, trunk position and stability, paw placement, and tail position.

The analysis shows that the control group recovered from damage slowly, improving by 1.357 BBB score values/week, compared to the (+)-EC group, which showed improved mobility at 4.45 BBB score values/week. The two-way ANOVA analysis showed a positive and significant (*p* = 0.006) treatment factor.

The molecular analysis was based on the work of Zhang [32]. We analyzed all molecules at the end of the experimental period in three sets of processes: (1) angiopoietin-1 as a marker of vascular changes; (2) beclin-1, myelin basic protein and glial fibrillary acidic protein as markers of cord degenerative processes and glial scar formation, and; (3) neurofilament FH and NeuN as markers of lost neurons.

In this sense, traumatic SCI disrupts the blood–spinal cord barrier and reduces the blood supply through microvascular changes. Vessel regression and neovascularization have been observed in secondary injury, contributing to microvascular remodeling after trauma. We analyzed vascular changes by measuring angiopoietin-1 levels. This vascular growth factor plays an important role in vascular stabilization and angiogenesis [33]. Reports suggest that its expression is reduced after spinal cord injury [34]. Ang-1 complements the VEGF system, contributing to endothelial cell survival and vessel remodeling. Some attempts have been made to give angiopoietin after SCI to increase angiogenesis and SCI recovery [35]. Our results showed that the traumatic injury induced a decrease in Ang-1 even three weeks after the trauma. Interestingly, Ang-1 levels in the (+)-EC-treated group remained similar to those in the sham group, suggesting that the flavanol can block deleterious vessel damage.

Interestingly, beclin-1 slightly but significantly increased three weeks after the traumatic SCI, and studies suggest that beclin-1 upregulation contributes to neuronal tissue damage [36]. These results might be controversial since it has been proposed that beclin-1-mediated autophagy protects against SCI [37,38,39]. We believe that our results align with those reported by Yin et al. [20], suggesting that autophagy inhibition protects the spinal cord against ischemic damage. Interestingly, (+)-EC blocked this increase, with the beclin-1 levels similar to those in the sham group.

Spinal cord injury also induces changes in myelin. This protein surrounds nerve cell axons to insulate them and modulates the electrical impulses that travel through the axon.

It has been reported that after SCI, MBP increases in the acute phase, returning to normal levels after seven days [40,41]. Our results show that MBP decreases three weeks after traumatic SCI; (+)-EC treatment blocks this decrease. The levels of this protein are slightly, but not significantly, increased compared to those of sham rats, suggesting that it facilitates remyelination.

On the other hand, the glial fibrillary acidic protein (GFAP) is affected by SCI. This protein, expressed in astrocytes, increases after spinal cord injury (SCI) and may serve as a biomarker of traumatic brain injury [40,41,42]. (+)-EC treatment blocks the TSCI-induced increase in GFAP, returning levels to those of the sham group.

In our experiments, NFH levels decreased and remained low three weeks after SCI, suggesting damage to neuronal axonal fibers. This protein is highly affected in neurodegeneration processes, particularly in spinal cord injury [42]. (+)-EC treatment blocks neuronal loss, as NFH levels remain comparable to those of the sham groups.

Cell differentiation and reduction of neuronal necrosis are other critical aspects of secondary damage following spinal cord injury. Neuronal nuclear antigen (NeuN) is a nuclear and perinuclear protein expressed in neurons in the central nervous system. Its decrease is associated with SCI [22]. Our results show a significant decrease in NeuN after TSCI, suggesting neuronal loss; interestingly, (+)-EC blocks neuronal loss.

TSCI requires long-term treatment. The cost of care and economic losses can affect the patients and their families, raising social and psychological issues. It is also clear that advances in understanding the molecular mechanisms of SCI are continuously growing. However, translating findings into effective clinical treatments remains challenging, and an active or permanent cure for this condition has yet to be developed. In this regard, several pharmacological approaches, including cyclooxygenase inhibitors [7], glutamate receptor antagonists [8], corticosteroids [9], neurotrophic agents [10,11], minocycline [12], and more, have been studied in clinical trials without precise results.

Although the results reported in the present work are remarkable and open up several possibilities for using (+)-epicatechin in the comprehensive analysis of mechanisms involved in the prevention and treatment of traumatic spinal cord injury, the results presented here have several limitations.

We did not directly address inflammatory responses or the presence of free radicals. However, we and others have reported that flavanols decrease inflammatory processes in several tissues and conditions [43,44,45] and that they can modulate oxidative stress, increasing the expression of internal modulators such as SOD and catalase [46,47,48,49,50]. We believe that (+)-EC affects these processes in this model.

We did not analyze the role of (+)-EC in the levels of excitatory amino acids, such as NMDA, or opioid receptors. However, even though we have demonstrated through in silico analysis that flavonoids can interact with this type of receptor, we lack direct proof of their modulation in this model. The results are also limited since we did not perform RNA sequencing (RNA-seq), proteomics, and immunohistochemistry (IHC) (e.g., GFAP/Iba1/NeuN co-staining to assess glial–neuronal crosstalk) to better understand the molecular mechanisms involved in the obtained results.

Our previous work showed that the epimer (−)-epicatechin binds selectively and with higher potency to the Apelin receptor [17] as compared to Apelin-13, that this epimer induces protection in a severe spinal cord injury model (complete spinal cord transection) [21], and that Apelin, an endogenous ligand of the G-protein-coupled Apelin receptor, is a potential therapeutic for central nervous system diseases by regulating autophagy, apoptosis, oxidative stress, and inflammation. In spinal cord injury, Apelin can inhibit the inflammatory response and optimize the surrounding microenvironment of the injured nerves [51]. It has also been proposed that the Apelin/Apelin receptor axis plays a relevant role in SCI since Apelin repairs the damage, regulating cell proliferation, apoptosis, and revascularization, improving motor function in the hindlimbs, and resulting in functional recovery after SCI [52]. We suggest that (+)-epicatechin could be acting through the binding to and activation of the Apelin receptor; this assumption needs more work to be proven. However, the results obtained with this flavanol warrant more complex experimental approaches to dissect the mechanism behind (+)-EC’s positive effects on protecting the spinal cord against trauma.

## 5. Conclusions

In conclusion, the results reported here demonstrate that the flavanol (+)-epicatechin provides significant protection against spinal cord injury resulting from moderate traumatic damage. These results strongly suggested that there was no progression of spinal cord damage after the injury. The (+)-EC-induced protection results in improved movement. Even when the results are clear and may serve as the basis for using (+)-epicatechin as a protective agent, further work is necessary to better understand the complete mechanisms involved in the obtained results. The results reported here warrant further investigation into the effects of (+)-epicatechin.

## Data Availability

Data will be made available upon the request.

## Supplementary Materials

The following supporting information can be downloaded at https://www.mdpi.com/article/10.3390/biom15060869/s1: Supplemental Video; Original Western blot images are provided in the Supplementary Materials.
