# Supplementary figures and images for: Positive Effects of (+)-Epicatechin on Traumatic Spinal Cord Injury Recovery
Source: Biomolecules. 2025 Jun 14;15(6):869. doi: 10.3390/biom15060869 (PMC12190840; doi:10.3390/biom15060869)

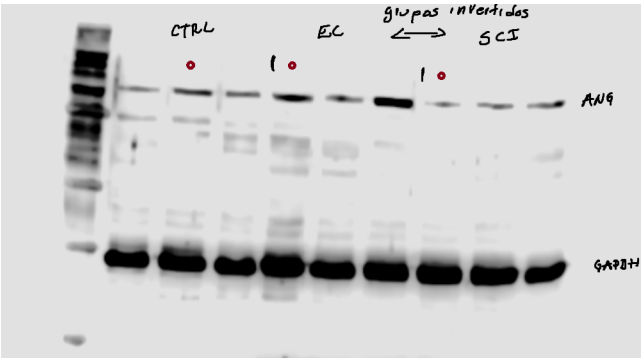

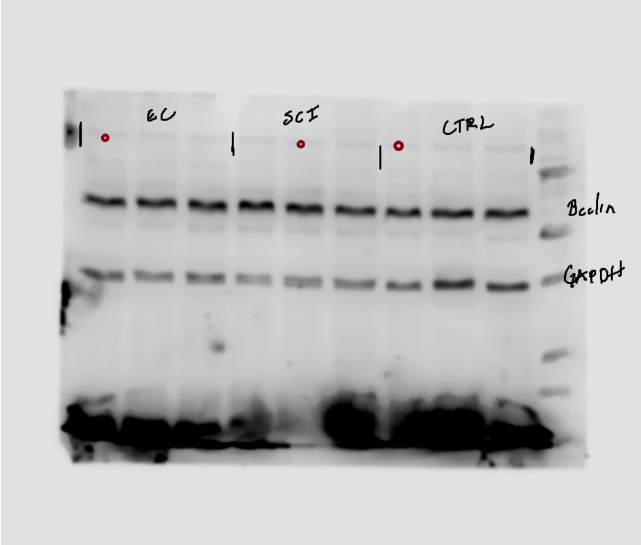

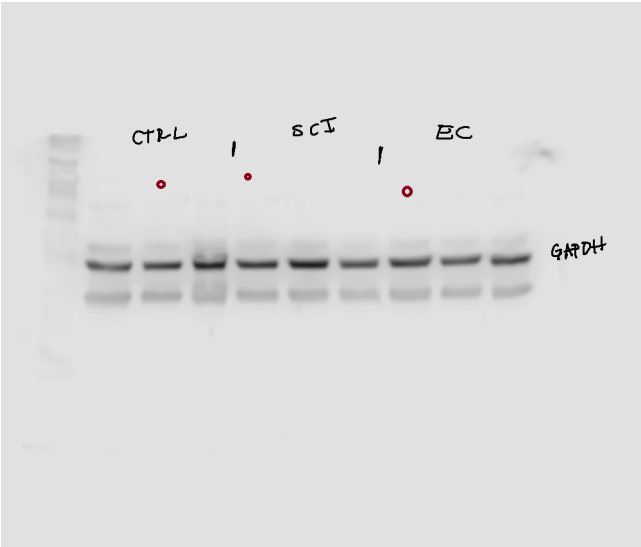

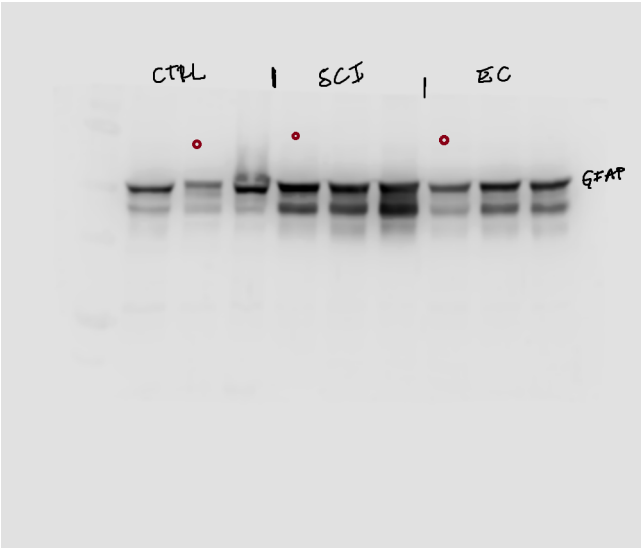

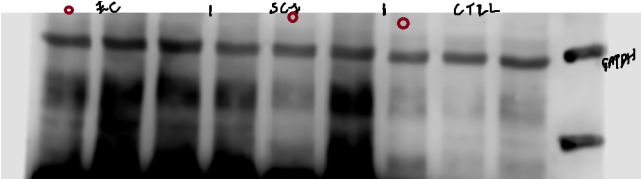

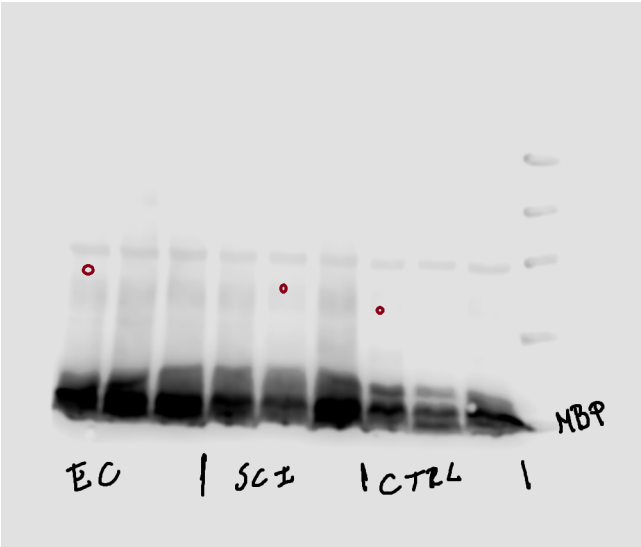

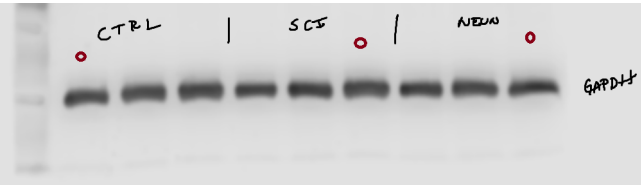

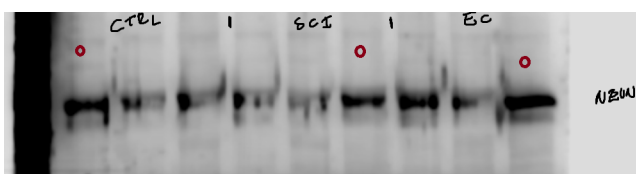

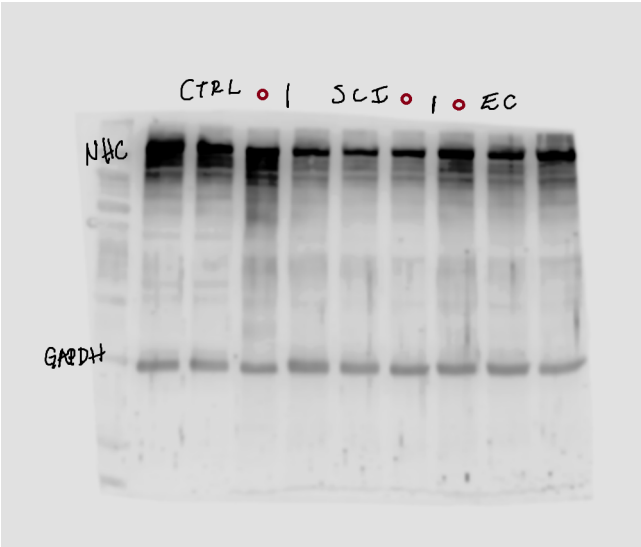

Supplement: Supplementary file 1 [file biomolecules-15-00869-s001.zip › biomolecules-3566706-WB.pdf]
